# Hierarchical Virtual Screening of Potential Insectides Inhibitors of Acetylcholinesterase and Juvenile Hormone from Temephos

**DOI:** 10.3390/ph12020061

**Published:** 2019-04-18

**Authors:** Glauber V. da Costa, Elenilze F. B. Ferreira, Ryan da S. Ramos, Luciane B. da Silva, Ester M. F. de Sá, Alicia K. P. da Silva, Cássio M. Lobato, Raimundo N. P. Souto, Carlos Henrique T. de P. da Silva, Leonardo B. Federico, Joaquín M. C. Rosa, Cleydson B. R. dos Santos

**Affiliations:** 1 Postgraduate Program in-Network in Pharmaceutical Innovation, Federal University of Amapá, Macapá, AP 68902-280, Brazil; vilhenac@hotmail.com (G.V.d.C.); elenilze.batista@ueap.edu.br (E.F.B.F.); 2 Laboratory of Modeling and Computational Chemistry, Department of Biological and Health Sciences, Federal University of Amapá, Macapá, AP 68902-280, Brazil; ryanquimico@hotmail.com (R.d.S.R.); luciaanebarros@hotmail.com (L.B.d.S.); 3 Laboratory of Biotechnology in Natural Products, Department of Biological and Health Sciences, Federal University of Amapá, Macapá, AP 68902-280, Brazil; estersa07@gmail.com (E.M.F.d.S.); aliciakarine1@gmail.com (A.K.P.d.S.); cassio153bio@gmail.com (C.M.L.); 4 Laboratory of Arthropoda, Federal University of Amapá, Macapá, AP 68902-280, Brazil; nonatoiepa@hotmail.com; 5 Computational Laboratory of Pharmaceutical Chemistry, School of Pharmaceutical Sciences of Ribeirão Preto, University of São Paulo, São Paulo 14040-903, Brazil; tomich@fcfrp.usp.br (C.H.T.d.P.d.S.); lbfederico@usp.br (L.B.F.); 6 Department of Pharmaceutical and Organic Chemistry, Faculty of Pharmacy, Institute of Biosanitary Research ibs. GRANADA, University of Granada, 18071 Granada, Spain; jmcampos@ugr.es

**Keywords:** acetylcholinesterase, juvenile hormone, temephos, molecular docking

## Abstract

*Aedes aegypti* (Linnaeus, 1762; Diptera: Culicidae) is the main vector transmitting viral diseases such as dengue fever, dengue haemorrhagic fever, urban yellow fever, zika and chikungunya. Worldwide, especially in the Americas and Brazil, many cases of dengue have been reported in recent years, which have shown significant growth. The main control strategy is the elimination of the vector, carried out through various education programs, to change human habits, but the most usual is biological control, together with environmental management and chemical control. The most commonly insecticide used is temephos (an organophosphorus compound), but *Aedes aegypti* populations have shown resistance and the product is highly toxic, so we chose it as a template molecule to perform a ligand-based virtual screening in the ChemBrigde (DIVERSet-CL subcollection) database, searching for derivatives with similarity in shape (ROCS) and electrostatic potential (EON). Thus, fourty-five molecules were filtered based on their pharmacokinetic and toxicological properties and 11 molecules were selected by a molecular docking study, including binding affinity and mode of interaction. The L46, L66 and L68 molecules show potential inhibitory activity for both the insect (−9.28, −10.08 and −6.78 Kcal/mol, respectively) and human (−6.05, 6.25 and 7.2 Kcal/mol respectively) enzymes, as well as the juvenile hormone protein (−9.2; −10.96 and −8.16 kcal/mol, respectively), showing a significant difference in comparison to the template molecule temephos. Molecules L46, L66 and L68 interacted with important amino acids at each catalytic site of the enzyme reported in the literature. Thus, the molecules here investigated are potential inhibitors for both the acetylcholinesterase enzymes and juvenile hormone protein–from insect and humans, characterizing them as a potential insecticide against the *Aedes aegypti* mosquito.

## 1. Introduction

*Aedes aegypti* (Linnaeus, 1762; Diptera: Culicidae) is the main vector transmitter of viral diseases such as dengue, hemorrhagic dengue, urban yellow fever, zika and chikungunya. The development of an effective vaccine against the four dengue serotypes is under study, but, until then, the estimate is that we will reach 390 million dengue infections per year, to which approximately 40% of the world population is potentially exposed. In addition, more than 200,000 cases of yellow fever have been registered each year [1,2,3]. In the Americas and Brazil, in particular, many cases of dengue have been reported in recent years, which have shown significant growth. The control strategy is the elimination of the vector, carried out through various educational programs and changes to human habits, but the most common approach is biological control, together with environmental management and chemical control. The insecticides used, besides being toxic to other living beings and the environment, suffer resistance by insect populations, so it is necessary to design and develop novel compounds that have a more selective and specific efficiency to the insect but to not cause problems to the host. The most common mechanism of action of commercial insecticides is the inhibition of the enzyme acetylcholinesterase [1,4,5].

Another mechanism of action includes juvenile hormones that, together 20-hydroxyecdysone, are pleiotropic, and their balance, since juvenile hormone needs a hemolymph index. These hormones determine the development of the insect, mainly acting in the growth process, controlling critical physiological events, performing repairs, being an important manager of the insect metamorphosis, also playing a role in reproductive (ovary) maturation in adults [6].

Treatments such as vaccines and specific drugs to prevent these mosquito-borne diseases (dengue, Zika) are not yet available so the design of insecticides with selectivity and specificity for insects is necessary for development of a compound without toxic effect to other living beings as well as the environment, since most of the commercial insecticides, due to their frequent use, suffers resistance by *Aedes aegypti* [7,8].

**Figure 1:**
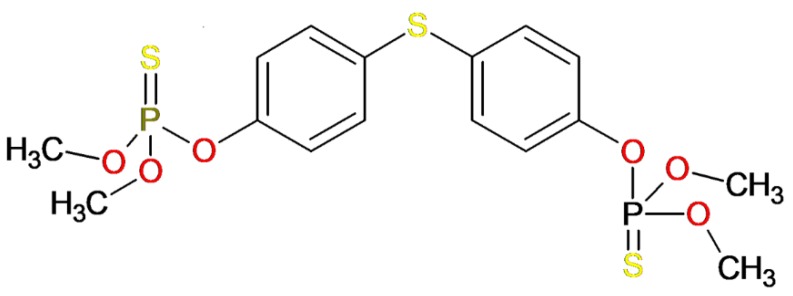
2D-structural formula of the temephos molecule (O,O,O′,O′-tetramethyl-O,O′-sulfanediylbis(1,4-phenylene)).

Organophosphate compounds like temephos have a characteristic phosphoric ester group in their chemical structure (Figure 1), which is still among most used class of functional groups in therapeutic agents, agricultural chemicals, plasticizers, flame retardants, fuel additives and, more notoriously, as chemical warfare agents. Since the 1970s tempehos is the main larvicide used to control the *Aedes aegypti* mosquito and is recommended by the WHO in drinking water at low concentrations. It has rapid action, however, it is quite persistent in the environment [9].

In this study, we carried out a design of molecules derived from temephos with potential insecticidal action against *Aedes aegypti* via ligand-based virtual screening, using the ChemBrigde (subcollection DIVERSet-CL) database, searching for molecules that share structural and electronic similarity with a template, using ROCS and EON software, and filtering the results by considering the pharmacokinetic and toxicological properties (with the Qikprop and DEREK softwares) as well as molecular docking. 

## 2. Results and Discussion

### 2.1. Empirical Force Field Methods (Molecular Mechanics–MM)

There is no crystallographic structure of the temephos molecule in a complex with the biological targets here investigated deposited in the Protein Data Bank (PDB), and it was necessary to optimize the molecule for a likely binding position, before performing the molecular comparison in the database to be screened. The molecular mechanics method was chosen for this optimization, since with such a method we could quickly obtain relatively precise geometric conformations [10,11]. The temephos molecule was optimized and its extended and low-energy conformation was used as a template structure to start the ligand-based virtual screening.

### 2.2. Hierarchical Virtual Screening

The virtual screening consists of structural analysis of the template molecule in regions of the conformational space and energetic hypersurfaces delineation of the molecule searched in a database, as well as in the active site of the macromolecule through molecular docking. It is a suitable method for the discovery and planning of new bioactive molecules [12]. The ChemBrigde database (sub-collection DIVERSet_CL) [13], containing about 480,000 compounds, was selected using the ROCS software to generate and classify three-dimensional structure overlaps that had only the conformation, fingerprints and a high level of precision of the structure of the temephos molecule being used as the first screening step to identify the top 2000 compounds

#### 2.2.1. Rapid Overlay of Chemical Structures (Using ROCS)

The temephos molecule was used as a template for comparison with each of all the molecules in the ChemBrigde (subcollection DIVERSet_CL) database, looking for chemical similarity, according to structural features and fraction of molecular volumes of the template, observing the maximum overlap with respect to the shape, according to the Gaussian function of smooth surfaces centered on the atoms [14,15]. Compounds were screened and sorted using an algorithm that generated scores relative to the database overlays, thus obtaining the 2000 top-ranked compounds that show highest scores and overlaps as well. After, selection of the molecules was performed according to their electrostatic potentials. ROCS aligns the molecules to provide a better input to the EON program [16].

#### 2.2.2. Electrostatic Similarity (Using EON)

The 2000 best ranked compounds as indicated by ROCS were then used as input for analysis using EON software, from which the 100 best molecules (top 100) of the ChemBridge_CL database were selected. Borges et al. [16], who carried out an analysis addressing the performance of the commercial screening methods based on commercial ligands and the acetylcholinesterase (AChE) structure, using ROCS and EON, have identified their relevance in finding potential hAChE inhibitors. Following this criterion, the most promissor screened molecules were again filtered in terms of predictions of pharmacokinetic properties (ADME), using the QikProp software, and toxicological profile, using DEREK.

#### 2.2.3. Pharmacokinetic and Toxicological Properties

Medicinal chemistry has as main aim the development of drugs for human beings, however, our approach was to find, using ligand-based virtual screening, a novel compound that shows higher potential larvicidal than temephos. Our pharmacokinetic selection was designed to determine a molecule with physicochemical properties equal to or better than thse of the temephos molecule. According to Burt [17], the mechanism of action of insecticides has two main steps to achieve a concentration that affects the central nervous system (CNS) of the insect. In the first stage the cuticle is divided into a biphasic system, comprising an external phase with lipophilic elements, and an internal phase of associated hydrophilic elements, and another pathway is hemolymph, which displays low penetration, so a alternative strategy used is via high penetration of the integument of the wall of the body, such as the trachea, becoming an important pathway for the CNS [18]. 

It has been observed that insecticides of the organophosphate class, like temephos, require a solvent to ensure better absorption in the body cavity of the insect [18]. Webb and Green [19], Hurst [20] and Läuger, Martin and Müller [21] have shown that insecticides whose molecules contain both an oil soluble group and a water soluble group, by orienting themselves suitably at an oil/water partition system, pass rapidly through the cuticle of the insect by diffusion along this interface. According to Matthews [22], the three major neurotransmitters found in both males and females are acetylcholine, glutamate and GABA. An ideal designed insecticide would be extremely toxic to *Aedes aegypti*, either by inhibiting acetylcholinesterase or juvenile hormones, but not to other species in the environment, although the toxicity will depend on the physiological and biochemical differences of the organisms to which they are targeted [5]. The pharmacokinetic properties that characterize the parameters required for a good insecticidal action are described in Table 1.

The parameters used for screening were established in the QikProp environment, and thus previously selected for using the Derek software, for searching molecules in the database with potential biocidal effect, in particular for *Aedes aegypti*, and not active against any other organism or the environment. Parameters were used to evaluate if the molecule can overcome the barriers and reach the CNS (Central Nervous System), so that the “star” parameter or drug-like properties were analyzed, which are compared with 95% commercial and common drugs [23]; MW, corresponding to the molecular weight of the molecule in (g·mol^−1^), ClogP/w, which corresponds to the octanol and water partition coefficient; HBD means hydrogen bonds that would be donated to the water solvent; HBA means the number of hydrogen bonds that would be accepted from a solvent [24,25,26,27,28]. The temephos molecule and those selected from the database were within the recommended values regarding the drug-like as well as the physicochemical properties, in order to show potential CNS action.

Another parameter available in QikProp is the biological prediction that shows the same focus of the study, i.e., the passage of the molecule to reach the enzymes under study: BB log, which establishes if the drug can overcome the blood/brain barrier, and MDCK, apparent cellular permeability, in nm/s, of two Madin-Darby canine kidney cell lines. MDCK cells are considered a good model for the blood-brain barrier [29,30]; Lipinski’s Rule of 5 predicts the most probable absorption and permeation rates [31]. Following our hieratic ligand-based virtual screening, 11 compounds were selected according to the criteria and parameters mentioned above. According to Voutchkova et al. [29], who used some QikProp parameters based on drugs that had not been evaluated in the EPA Toxic Release Inventory (TRI) reference parameters, correlating the toxic effect with recommended values of commercial drugs, a selection was performed in the present work. The temephos molecule showed the expected values for the toxic molecule and also showed a high value as recommended by TRI, confirming its high toxicity, and our molecules are also within the recommended TRI standard, as observed in Table 1.

The screening parameters were established through the QikProp program that facilitated the filtering in the DEREK software, searching for database molecules with potential biocidal effects and no toxic action in any other organism or the environment as well. The temephos molecule in the DEREK program, obtained as prediction cholinesterase inhibition, hepatotoxicity and skin sensitization. None of the 11 molecules analyzed showed any kind of plausible toxic alert.

According to the WHO [28], temephos can be used to control mosquitoes in potable water, but should not exceed an amount of 1 mg/L. Temephos was tested for toxicity and mutagenicity against *Escherichia coli* and *Salmonella typhimurium*, and at concentrations above 3.33 μM it was mutagenic and genotoxic, with and without metabolic activation [32]. In a study by Lee with fish of the species *Fundulus heteroclitus*, it was found that temephos was the compound that presented the highest acute toxicity among the compounds analyzed at a concentration of 0.04 mg/L [33]. A toxicity study also conducted on survival and subsequent emergence of *Arnitus hesperidurn* and *Prospaltella opulenta* found no toxic effect of the 14 insecticides evaluated along with temephos, but correlated that a high solubility of compounds above 2000 ppm in water directly influences the life of the species analyzed [34].

A direct relationship between the degree of AChE inhibition and toxicity might not always be expected [35]. A study was conducted to determine the median lethal concentration of an emulsifiable formulation of temephos as well as the response of BChE and AChE enzymes and to study their effects on cholinesterase behavior and activity in green frog tadpoles (*Rana clamitans*). A concentration of 10 μL/L caused death of all the subjects, but there was no favorable expression in respect to the enzymes [36]. A more complete study carried out by Junges [37] investigated the lethal and sublethal effects of three insecticides (temephos, *Bacillus thuringiensis* var. Israelensis and permethrin) on amphibians (*Rhinella arenarum, Rhinella fernandezae* and *Physalaemus albonotatus*) and temephos was the second most toxic and all insecticides were observed to produce behavioral changes in tadpoles independent of the dose-response. The toxic effects of temephos in cichlid fish (*Tilapia melanopleum*) and dragonfly larvae (Odanata) *Neurocordulia virginiensis*, which established a estimated safe concentration for the fish of 3.0 mg/L, were analyzed in the renewal toxicity test, giving a results of 0.2 mg/L for insect larvae [38].

Despite many studies about toxicity of temephos, the degree of expected adverse effects on living beings as well as the environment remains inconclusive. For the ligand-based virtual screening approach here used for selection of molecules with potential activity in the CNS of *Aedes aegypti*, molecular docking for only the best-ranked and selected 11 molecules was performed, in order to complete our study with the design of molecules that could minimize or cause no affects to other species or the environment, but with specific biocidal action in inhibiting the acetylcholinesterase and juvenile insect hormone enzymes.

#### 2.2.4. Molecular Docking Procedures

**Figure 2:**
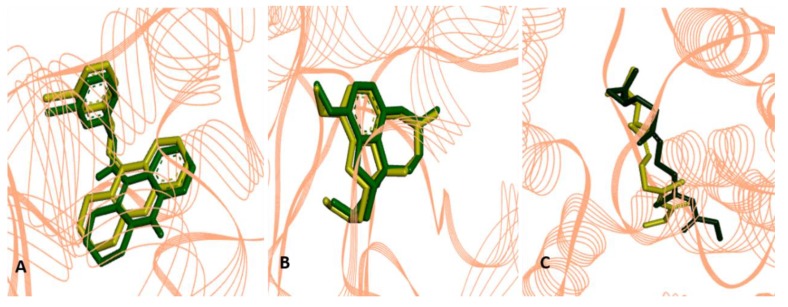
Overlays of crystallographic ligands poses: in (**A**) I40 (in green), with the calculated pose (in yellow), in (**B**) GNT (in green) with the calculated pose (in yellow), and in (**C**), JHIII (in green) with the calculated pose (in yellow).

Molecular docking was performed using a validation protocol in order to determine the pose (conformation + orientation) of the ligands (I40, GNT and JHIII) inside the enzyme active sites (PDB IDs 1QON, 4EY6 and 5V13, respectively, to the mentioned ligands order). The mean square root deviation (RMSD) between the reference ligands and the experimental ligands of I40 (0.85 Å), GNT (0.34 Å) and JH31 (1.33 Å) were calculated. The RMSD analysis was determined by considering the most adequate position of the initial structure around the X-ray crystallographic complex, analyzing experimentally the most stable position so that there is no structural change between the proteins and the reference ligand when complexed with the macromolecule under study. The best molecular fit is determined by an RMSD less than or equal to 1.5 [39,40,41], as shown in Figure 2.

According to Harel [42], a study with a potent inhibitor of the enzyme acetylcholinesterase called I40 for *Drosophila melanogaster* (PDB ID 1QON) was conducted, noting that interactions occur mainly between Trp-83 and, Trp-472, Phe-330, Tyr-71, around of the α-helix between the amino acid residues Tyr-370 and Tyr-374. He also specified that the active site modifications occur in nine residues in the throat of the active site (Tyr-71, Trp-83, Tyr-324, Phe-330, Tyr-370, Phe-371, Tyr-374, Trp-472 and His-480). 

The active site of the human AChE has an extension of 20 Å, with a catalytic active site (CAS) and a peripheral anionic site (PAS). Since the functionality of AChE’s active site depends on the specificity of the catalytic triad of amino acid residues Ser-203, His-447 and Glu-334, it involves reactions of substrates and catalyze the hydrolysis of acetylcholine in acetic acid and choline, by an oxyanion orifice consisting of Gly-121, Gly-122 and Ala-204. In the anionic subsite, site-directed mutagenesis indicates that the aromatic residues Trp-86, Trp-9, Trp-10, Glu-202 cationic moiety is often attributed to being electrostatic and Phe-337 plays an important role in terminal trimethylammonium attachment [43,44,45].

The first step to initiate molecular docking is to perform binding affinity analysis of all the ligands deposited in the PDB and the ligands under study, i.e., the acetylcholinesterase inhibitors of the insect *Drosophila melanogaster*, fruit fly (PDB ID 1QON, I40), human acetylcholinesterase (PDB ID 4EY6, GNT) and juvenile homone (PDB 5V13, JHIII), for interactions that show higher binding affinity than the specific ligand (I40, GNT), but for JHIII the L46, L66 and L68 ligands results were better than for the enzyme refiner ligand. 

After analysis of the 11 compounds submitted to docking, only the ligands L46, L66 and L68 showed higher values than those observed for temephos, our template. The ligands did not show any of the characteristic features of the temephos molecule, such as the phosphorus atom, with a double bond to the sulfur. However, molecules L46, L66 and L68 demonstrated excellent binding affinity and interaction with the enzyme acetylcholinesterase, higher than that found for the temephos molecule. Validations were used as parameters for molecules that are complexed in the PDB, which do not present such groups and have effectiveness in the inhibition of the enzyme acetylcholinesterase according to Srivastava et al. [46], who performed design and development of some phenyl benzoxazole derivatives as potent acetylcholinesterase (AChE) inhibitors by in vivo and ex vivo analyses, and revealed the true nature and competitive type of the AChE inhibition among their analyzed molecules, even though they did not have a phosphorus atom and sulfur neither.

**Figure 3:**
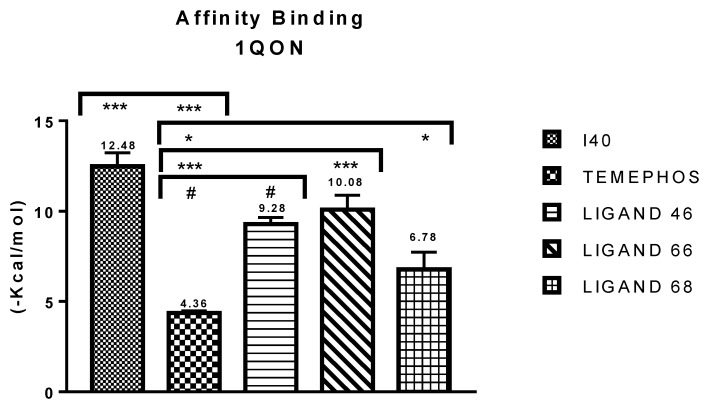
Results of binding affinity of the compounds with insect acetylcholinesterase receptor.

The binding affinity indicates a strong bond when it is the lowest, signaling that designed ligands will have excellent interaction with the receptor [47,48]. The binding affinity of the analyzed molecules in the macromolecule PDB ID 1QON of the fruit fly did not present values higher than I40 (−12.71 kcal/mol), but all the ligands evaluated presented values adapted *p* <0,0206, in respect to temephos (−8.11 kcal/mol), which showed a difference of −4.92, −5.72, −2.42, L46, L68 and L66, respectively. Compound L66 (−10.68 Kcal/mol) had a value closer to I40 with a significant difference of only 2.4, followed by L46 (−10.96 kcal/mol), with a significant difference of 3.2, according to Figure 3.

**Figure 4:**
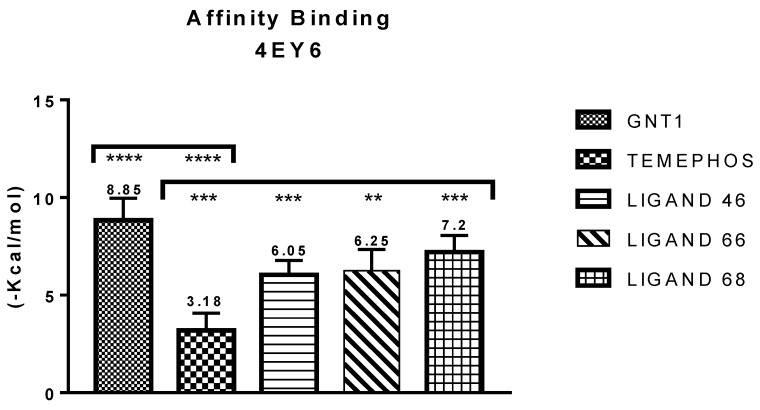
Results of binding affinity of the compounds with human acetylcholinesterase.

For human acetylcholinesterase (PDB ID 4EY6) the ligands showed high binding affinity when compared to the controls used in the molecular docking study (temephos), values that approximate the GNT binding affinity values (−9.72 kcal/mol), whereas the ligands show −6.25 kcal/mol, ligand 46 shows −6.05 Kcal/mol and temephos shows −3.18 kcal/mol, each one showing a difference of 1.65, 2.6, 2.8 and 5.63. Compound L68 shows −7.2 Kcal/mol, a value close to the found for GNT, according to Figure 4.

**Figure 5:**
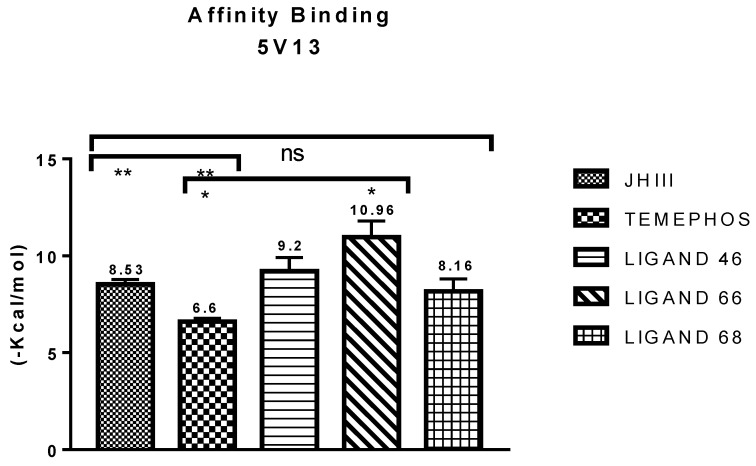
Results of binding affinity of the compounds with the juvenile hormone receptor (PDB ID 5V13).

In order to carry out the molecular docking with PDV 5V13, the ligands L46, L66 and L68 were used, even though the ligands had values higher than temephos or JHIII. The results of the affinity values can be observed in Figure 5.

Binding affinity of the compounds here investigated have shown values higher than those observed for JHIII (−8.53 kcal/mol) and temephos (−6.6 kcal/mol), whereas L46 ligands (−9.2 kcal/mol), L66 (−10.96 kcal/mol) and L68 (−8.16 Kcal/mol) presented adjusted values (*p* <0.0001), in respect to the two molecules. Compound L66 shows a value above that found for the juvenile hormone ligand (JHIII), with a significant difference of −2.43, followed by L46, with a significant difference of −0.67, and there was no significant difference in respect to the L68 molecule, however all the ligands had equal or higher binding affinity than to JHIII and temephos.

**Figure 6:**
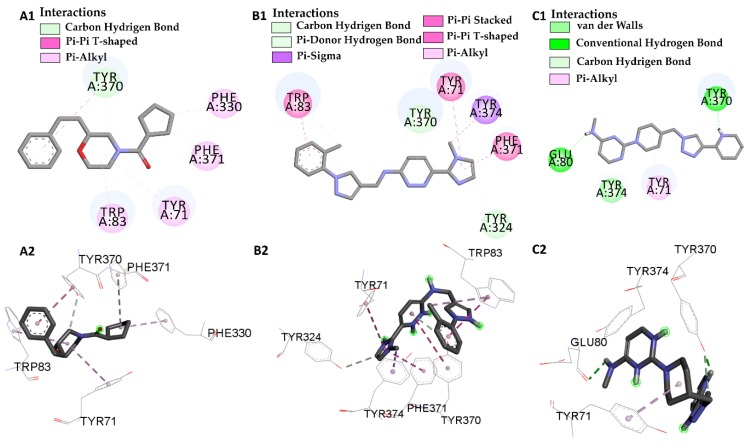
Interactions of the active site of the insect acetylcholinesterase (Drosophila melanogaster) with the molecules L46 2D (**A1**) 3D (**A2**), molecules L66 2D (**B1**) 3D (**B2**) and molecules L68 2D (**C1**) 3D (**C2**).

Results found after the molecular docking was in accordance with the reported by Harel [42], where the PDB ID 1QON macromolecule and I40, L46, L66 and L68 ligands showed similar interactions between all ligands and the β-sheet containing Trp-83 and the α-helix between amino acid residues Tyr-370, Tyr-374 (except L46), Tyr-71 (except L66), confirming our selection for a potential inhibitor of the insect acetylcholinesterase enzyme. Compounds L46 and L66 showed the same interactions observed for compound I40, indicating strong interaction with the acetylcholinesterase catalytic site, around the helix located between amino acid residues Trp-83, Tyr-370, Tyr-71, Phe-371, see Figure 6.

Organophosphates are insecticides that have as mechanism of action the ability to irreversibly inhibit the enzyme acetylcholinesterase, which acts directly in the post-ganglionic synaptic cleft, and has as a function the elimination and reuptake of the neurotransmitter acetylcholine, so that there is no excess of nerve impulse and cell death. In addition many substrates are hydrolyzed through a nucleophilic attack generating an acyl enzyme or a phosphoryl-enzyme intermediate and then deacylation or dephosphorylation, becoming an irreversible reaction [35,49].

Compounds L46, L66 have interactions with Ser-203 and L66 and L68 with His-447, showing that our ligands were able to interact only with two amino acids of the AChE catalytic triad, residues Ser-203, His-447, but did not interact with Glu-334. However, Glu-202 appeared in our study, which is also part of the amino acid residues with modification in the active site enzyme, analyzing interaction with ligands L66 and L68 and also with the galatamine refining ligand. The main mechanism of inhibition involving the amino acid residue Ser-203, in which it covalently binds between the central phosphorus side chain [50]

According to Han et al. [45] who performed a study of potent inhibitors of the enzyme acetylcholinesterase analyzing nine residues (Tyr-72, Tyr-124, Tyr-341, Tyr-286, Phe-295, Phe-297, Trp-236, Tyr-337 and Phe-338) with potential modifications in the enzyme and possible inhibition, L66 ligands showed interactions with Tyr-124, Phe-338, L46 interacted with Trp-236 and all ligands under study demonstrated interaction with Tyr-337.

**Figure 7:**
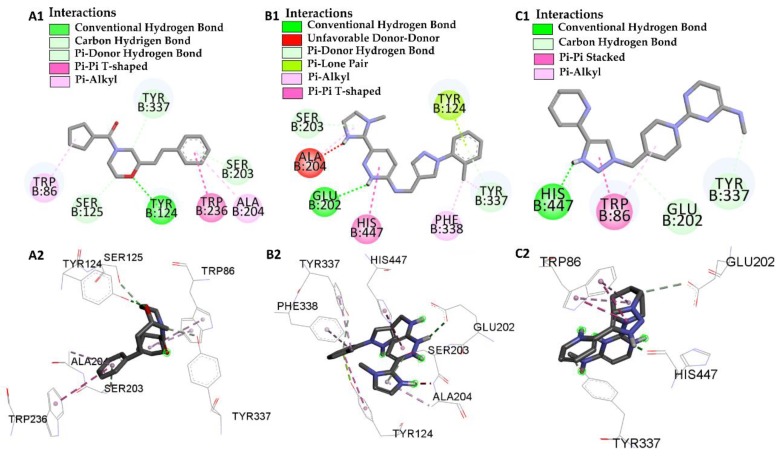
Interactions of the human acetylcholinesterase active site from *Homo sapiens* with molecules L46 2D (**A1**) 3D (**A2**), molecules L66 2D (**B1**) 3D (**B2**) and molecules L68 2D (**C1**) 3D (**C2**).

Interactions were observed for compounds L46, L66 and L68 at the catalytic site of the enzyme, confirming the binding affinity values, and thus achieving a better inactivation of the enzyme and allowing them to be considered as potential inhibitors of the enzyme acetylcholinesterase with insecticidal action as well, even presenting a lower binding affinity value, see Figure 7.

**Figure 8:**
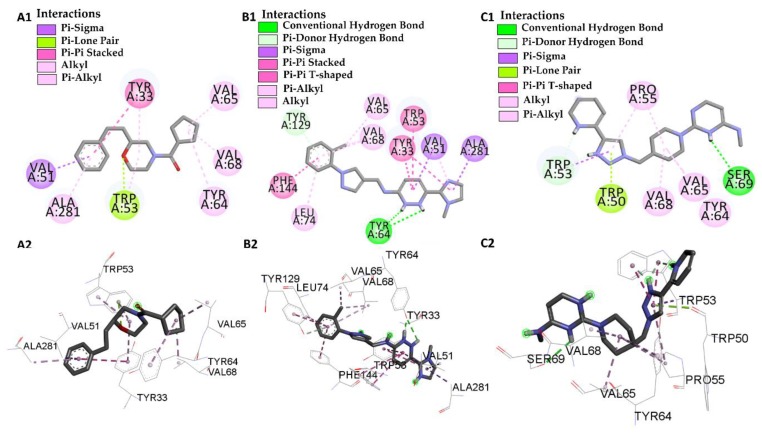
Interactions of the juvenile hormone active site from *Aedes aegypti* with the molecules L46 2D (**A1**) 3D (**A2**), molecules L66 2D (**B1**) 3D (**B2**) and molecules L68 2D (**C1**) 3D (**C2**).

The amino acid residues of the juvenile hormone protein interacting with JHIII in *Aedes aegypti* were described by Kim et al. [51], who determined that the epoxy group forms a hydrogen bond with the phenolic hydroxyl of Try-129, and we can observe that the ligand L66 shows this interaction and the rest of the isoprenoid chain is surrounded by hydrophobic side chains including those of Phe-144, Try-64, Trp-53, Val-65, Val-68, Leu-72, Leu-74, Val-51 and Tyr-33. The L66 ligand did not only show an interaction with Leu-72, but could also be an excellent modulator of the juvenile hormone protein in its active site, followed by the ligands L46 and L68 which have shown interactions with Try-64, Trp-53, Val-53, Val-65, Val-68, whereas interactions with Val-51 and Try-33 were found only on L46, with protein modification, see Figure 8.

The juvenile hormones, together with the 20E, are pleiotropic molecules produced by the corpus allatum, that need to maintain their homestasi. As an example, the juvenile hormone must maintain a certain index in the hemolymph, so that the insect displays adequate growth during the phases of its development. The hormones determine the development of the insect, acting mainly in its growth process, controlling critical physiological events, performing repairs, being an important insect metamorphosis manager, and play a role in reproductive maturation (ovary) in adults [6,52].

The L66 molecule show higher binding affinity than the L68 molecule, with several residues reported in the literature that may cause modification in the active site as a consequence of the enzymatic inhibition in the active site, for which the docking here performed show that the interactions mainly occur with Trp-83, Try-370, Phe-372 and Try-71—the same interactions encapsulated in I40—the ligand complexed in the PDQ ID 1QON. On the other hand, for the molecule L68, which shows a lower binding affinity and with only Try-370 and Try-374, similar to I40, the bond-free energy is very low, −1.33, which shows that factors such as the number of uncharged hydrogen bonds, the size of the polar or non-polar surface portions, the number of rotational bonds or the enthalpy required to desolvate the ligand or protein, are physicochemical parameters that may characterize a low free energy value [53]-see Table 2.

The molecules L46 and L66 form π-alkyl type interactions with Trp-83, similar to those that occur with the reference molecule - I40. The molecule L66 shows a very low free energy (−3.88 kcal/mol), but the interactions with Ser-203, at a distance of 2.93 Å, through a π-donor type bond with His-447, make interactions of π-π-stacked type with the Tyr residue, forming a π-alkyl type interaction similar to those occurring with GNT (see Table 3).

The L66 molecule shows a low ΔG (−3.88 Kcal/mol), however, it interacts with Ser-203, Glu-202, His-447, Tyr-124 and Tyr-337, mainly via π-alkyl bonds, with a mean distance of 3.45 A, and its Ki is 1.42 nM (see Table 4). With increasing temperature, the hydrogen bonding fuses and each interacting -CH contributes with about −600 cal/mol to the stability (ΔG) of the complex. The Van der Waals energy type bonds show a lower value for electrostatic energy because of the detailed interactions of the atoms with each residue of the protein-activator. In addition, the increased hydrophobicity of the amino acid residues and the loss of α-helix content (from 63.57 to 51, 83%) in presence of hydrogen bond acceptors, reveals their motif [54,55,56].

### 2.3. Structure-Activity Relationship of the Promising Molecule

The molecule L46 has a characteristic morpholine-4-carbaldehyde group which is synthesized and patented as a new amino derivative, and their use as a pharmaceutical refers to a class of dopamine agonists, more particularly a class of agonists that are selective for D3 over D2 [57]. Ligands 66 and 68 show a structure very similar to a novel class of insecticides, for example, pymetrozine, which has a similar structure to the ligands here investigated. Pimetrozime has a mechanism of action that is not yet fully understood, but it affects the nerves that control the salivary pump and causes immediate reactions and irreversible cessation of feeding due to an obstruction of stylet penetration, followed by hunger and death of insects, while another presumed mechanism selectively affects the cordotonic mechanoreceptors of insects [58,59,60,61].

**Figure 9:**
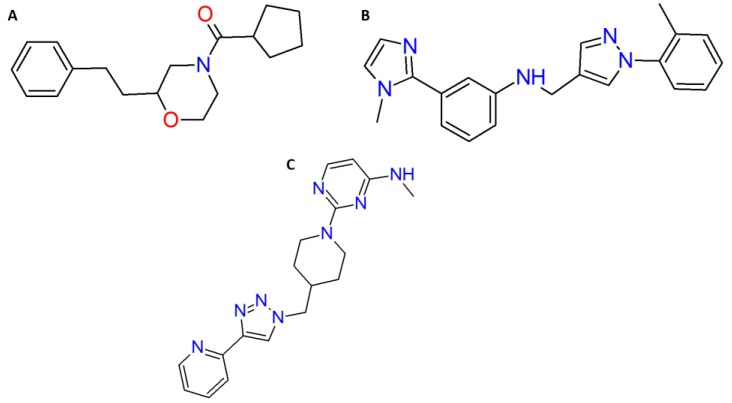
Promising molecules L46 (**A**) and L66 (**B**) and L68 (**C**), selected by virtual screening.

The ligands L46, L66 and L68 (see Figure 9) were tested via a high-throughput screening (HTS) assay based on primary cells, for identification of small molecule agonists of *Aedes aegypti* NPYLR7 [Small Molecule Inhibitors of Behavior of Mosquito Bites], a mouse cGAS RapidFire screen, human cGAS chemiluminescence screen, and the molecules were considered inactive in these analyzes [62,63,64,65].

## 3. Materials and Methods

### 3.1. Template Compound

The crystallographic pose of the temephos molecule is not available in any database, nor complexed with any macromolecule in the PDB, so that it was necessary to perform an energy minimization to select an extended low-energy conformation, before beginning the virtual screening based on ligands [10].

### 3.2. Generation of Conformers Library in Database

For discovery of potential new ligands susceptible to the temephos insecticide model, the following compound database was used: ChemBridge (subcollection DIVERSet-CL, (https://www.chembridge.com/screening_libraries/), which offers two complementary libraries with 480,000 compounds. For each molecule in the database, up to 300 conformers were generated using the MMFF94 force field implemented with the OMEGA software (Open Eye Scientific Software, Santa Fe, NM, USA, http://www.eyesopen.com), on a computer equipped with an Intel Core i7 2.4 GHz processor, using the Windows 7 Professional operating system. The strain energy (energy difference regarding the global minimum energy) of up to 9 kcal/mol, and a mean square deviation (RMSD) of 0.6 Å [66] were employed for non-redundant conformers selection.

### 3.3. Virtual Screening Procedures

#### 3.3.1. ROCS (for Shape Similarity)

In this project, the Rapid Overlay of Chemical Structures (ROCS) v2.4.1 software (OpenEye) was used as a tool for three-dimensional (3D) molecular similarity searches. The use of ROCS leads to the identification of a set of new compounds showing synthetic opportunities to further optimize biological affinity. In this shape-based overlapping method, the molecules are aligned by maximizing the overlap volume between a reference frame (temephos) and each molecule contained in the database, and after that they will be screened and selected using the algorithm to generate and rank the three-dimensional overlaps of the database with the reference structure (temephos).

#### 3.3.2. EON (for Electrostatic Similarity)

The EON software (for electrostatic similarity calculations) performs electrostatic comparisons, comparing electrostatic potential maps of pre-aligned molecules, and determines the Tanimoto indexes, for comparison. Classification of the structures is done using the "Electrostatic Tanimoto Combo Score" (ET_combo) which is the sum of the Tanimoto Poisson-Boltzmann (ET_pb) electrostatic coefficient and the Tanimoto shape (ST). EON does not overlap or change the orientation of the structures used. Therefore, since the alignment used in both programs is the same, similar performances in the metrics can be expected [67].

#### 3.3.3. In Silico Pharmacokinetic and Toxicological Properties

For the analysis of the pharmacokinetic properties, QikProp was used, which is a fast and accurate software for prediction of physicochemical properties such as absorption, distribution, metabolism and excretion (ADME) and presents ranges of comparison between the properties of the molecule or compound being analyzed with 95% of the drugs known and used as reference [25,26]. Toxicity profile analysis of the compounds was evaluated using the Deductive Estimation of Risk from Existing Knowledge (DEREK) software. This software makes qualitative predictions and, in this way, generates warnings about the possible toxic action of the chemical compounds analyzed by it. The system is able to interpret toxicophoric substructures present in the compounds as possible inducers of certain types of toxicity–the endpoints [16,63].

#### 3.3.4. Molecular Docking Simulations

##### Selection of the Proteins and Ligands Structures

Two macromolecules of the acetylcholinesterase enzyme were used, (PDB 1QON) and one from humans (PDB 4EY6). The crystallographic structure of acetylcholinesterase (AChE) from *Drosophila melanogaster* in complex with with tacrine [(9-3-iodobenzylamino)-1,2,3,4-tetrahydroacridine] (I40) was used, solved by X-ray diffraction and resolution of 2.7 Å [53]. The crystallographic structure of the recombinant human acetylcholinesterase (hAChE) complexed with (–)-galantamine (GNT), solved by X-ray diffraction and resolution of 2,4 Å [27] was also used. The crystallographic structure of juvenile hormone (PDB ID 5V13), in complex with (2E,6E)-9-[(2R)-3,3-dimethyloxiran-2-yl]-3,7-dimethylnona-2,6-methylene dienoate, (JHIII), with resolution of 1.87 Å [16] was also used. I40, GNT and JHIII and pyriproxyfen were used as control ligands in the molecular docking study based on the standard protocol established by our research group [9,68,69,70].

##### Docking Study with AutoDock 4.2/Vina 1.1.2 via Graphical Interface Pyrx (Version 0.8.30)

In order to validate the molecular docking method, the compounds with the crystallographic information were submitted to the development of docking until the spatial conformation was found through the AutoDock 4.2/Vina 1.1.2 [71,72] software through the Pyrx interface, by comparison with the original crystallographic structure of the acetylcholinesterase compound. PDB IDs 1QON and 4EY6 and juvenile hormone III (PDB ID 5V13) were used. Both the ligands and protein structure used in molecular docking were prepared using the Discovery Studio 5.0 software, whereas AChE (from *D. Melanogaster* and *Homo sapiens*) in complex with inhibitors as well as the juvenile hormone (from *Aedes aegypti*) ligands were used in AutoDock 4.2/Vina 1.1.2 and Pyrx interface (version 0.8.30), respectively. Validation of the molecular docking of the ligand was performed by comparing the pose of the crystallographic inhibitor and the docking pose obtained for the same inhibitors (structures of PDB IDs: 1QON, 4EY6 and 5V13), based on the RMSD value, (see Table 5).

##### Molecular Binding Affinity Statistics

In order to evaluate the affinity of the compounds, the data at each position calculated by the Pyrx software were typed in an Excel spreadsheet and analyzed by the One-way ANOVA followed by Turkey test (5%) multiple comparisons test was performed using GraphPad Prism version 7.00 for Windows, GraphPad Software, La Jolla, CA, USA, www.graphpad.com.

## 4. Conclusions

Design of a novel potential biocide that had a superior effect to temephos was achieved through ligand-based virtual screening. We selected molecules with potential insecticidal activity with a likely mechanism of action similar to the template molecule (temephos), with very selective and specific pharmacokinetic and toxicological properties that do not affect the environment or other living beings.

The most promising molecules L46, L66 and L68 were shown to be inactive in *Aedes aegypti* primary cell analyses, how also, the mouse cGAS RapidFire, human cGAS chemiluminescence screen. However, in our study promising molecules showed excellent results for both the acetylcholinesterase as well as to the juvenile hormone receptor, performing inhibition by these two mechanisms of action on *Aedes aegypti*. Therefore, the inhibitor-enzyme interactions of these molecules were similar and superior to those observed for the reference compounds (GNT, JHIII), as well as the template molecule (temephos). Our research group intends, in future works, to build an AChE model for *Aedes aegypti* through homology modeling studies, as well as to carry out biological assays with the molecules obtained in this work, in order to confirm such in silico predictions.

## Figures and Tables

**Table 1:** Pharmacokinetic properties of selected molecules.

Molecules	ID	Star ^a^	CNS ^b^	MW ^c^	ClogP/*w* ^d^	log (BB) ^e^	MDCK (nm/s) ^f^	HBD ^g^	HBA ^h^	R5 ^i^
Normal range	-	0–5	−2 to +2	130–725	−2.0 to 6.5	−3.0 to 1.2	<25 poor, >500 great	0–6	2–20	Max. 4
TRI (toxic) compounds ^l^	-	-	-	-	−2.1 to 6.99	−1.67 to 0.794	25.65 to 10^4^	0–3	0–9.53	-
Temephos	-	5	0	466.458	7.333	−0.491	10^4^	0	3.00	1
omega_22273_1_1_66	L66	0	0	345.406	4.251	−0.430	947.552	1	5.00	0
omega_23792_1_1_68	L68	0	0	350.425	3.507	−0.656	563.417	1	6.50	0
omega_32859_1_1_12	L12	0	1	315.429	4.163	0.269	3898.807	0	3.75	0
omega_5087_1_1_46	L46	0	1	287.401	2.931	0.079	3128.836	0	4.70	0
omega_36855_1_1_257	L257	0	0	372.441	3.550	−0.423	1578.497	0	7.20	0
omega_31303_1_1_291	L291	0	0	348.404	3.560	−0.644	538.073	1	6.25	0
omega_20865_1_1_102	L102	0	1	282.401	2.380	−0.049	3166.685	0	4.70	0
omega_12589_1_1_18	L18	0	0	381.474	3.147	−0.425	696.935	0	7.50	0
omega_45720_1_1_22	L22	0	0	339,317	3.894	−0.052	3333.563	1	5.70	0
omega_28697_1_1_35	L35	0	0	354.451	2.310	−0.581	620.602	0	7.50	0
omega_34836_1_1_50	L50	0	0	341.452	2.661	−0.581	648.835	1	6.50	0

^[a]^ Number of computed properties which fall outside the required range for 95 % of known drug; ^[b]^ Activity in the central nervous system; ^[c]^ Molar weight; ^[d]^ partition coefficients for octanol/water; ^[e]^ Predicted brain/blood partition coefficient ^(f)^ cellular permeability, in nm/s, of two cell linesdMadin-Darby canine kidney (MDCK) cells (Affymax scale) ^(g)^ Number of hydrogen bonds donated by the molecule; ^[f]^ Number of hydrogen bonds accepted by the molecule; ^[i]^ Number of violations of Lipinski’s ‘Rule of Five’.

**Table 2:** Interactions between the insect acetylcholinesterase and the most promising molecules.

Molecular Docking	Residues	Distance (Å)	Type	ΔG (kcal/mol)
I40 vs PDB ID 1QON	Trp-83	4.1/4.497	Pi-Alkyl	−12.66
	Trp-83	3.66/5.66	Pi-Pi Stacked	
	His-480	2.75	Conventional Hydrogen Bond	
	Phe-371	4.88	Pi-Alkyl	
	Try-370	3.79/4.18	Pi-Pi Stacked	
	Try-370	2.99	Pi-donor	
	Try-374	4.34	Pi-Alkyl	
	Try-71	4.49	Pi-Pi Stacked	
	Try-71	3.98	Pi-donor	
L46 vs PDB ID 1QON	Trp-83	5.00	Pi-Alkyl	−6.94
	Try-370	4.37	Pi-T-Stacked	
	Try-370	3.26	Carbon Hydrogen Bond	
	Try-71	5.41	Pi-Alkyl	
	Phe-330	4.39	Pi-Alkyl	
	Phe-371	5.41	Pi-Alkyl	
L66 vs PDB ID 1QON	Trp-83	4.41	Pi-Pi-Stacked	−9.55
	Trp-83	4.65	Pi-Alkyl	
	Try-370	5.98	Pi-Pi-Stacked	
	Try-371	4.01	Pi-Alkyl	
	Try-324	3.68	Carbon Hydrogen Bond	
	Try-374	3.89	Pi-Sigma	
	Phe-371	4.55	Pi-T-Stacked	
L68 vs PDB ID 1QON	Try-370	1.73	Convent. Hydrogen Bond	−1.33
	Try-370	3.32	Carbon Hydrogen Bond	
	Try-374	3.98	Pi-Sigma	
	Try-371	4.01	Pi-Alkyl	
	Glu-80	2.80	Convent. Hydrogen Bond	

**Table 3:** Interactions between the human acetylcholinesterase and the most promising molecules.

Molecular Docking	Residues	Distance (Å)	Type	ΔG (kcal/mol)
GNT vs PDB ID 4EY6	Trp-86	5.08/4.54/4.14	Pi-Alkyl	−9.72
	Ser-203	2.61	Conventional Hydrogen Bond	
	His-447	3.54	Carbon Hydrogen Bond	
	Glu-202	2.31	Conventional Hydrogen Bond	
	Tyr-337	5.45	Pi-Alkyl	
	Tyr-124	3.73	Carbon Hydrogen Bond	
L46 vs PDB ID 4EY6	Trp-86	4.30	Pi-Alkyl	−7.64
	Trp-86	5.31	Pi-Alkyl	
	Ser-125	2.78	Carbon Hydrogen Bond	
	Tyr-124	2.65	Conventional Hydrogen Bond	
	Trp-286	5.64	Pi-Pi-T-Shaped	
	Ala-204	5.18	Pi-Alkyl	
	Ser-203	2.78	Pi-donor Hydrogen Bond	
	Tyr-337	2.66	Carbon Hydrogen Bond	
L66 vs PDB ID 4EY6	Ser-203	2.93	Pi-Donor	−3.88
	His-447	5.54	Pi-T-Stacked	
	Tyr-124	2.91	Pi-T-Stacked	
	Tyr-124	5.63	Carbon Hydrogen Bond	
	Tyr-337	4.73	Pi-Alkyl	
	Tyr-337	3.43	Pi-Donor	
	Glu-202	2.72	Conventional Hydrogen Bond	
	Phe-338	4.88	Pi-Alkyl	
	Ala-204	4.09	Pi-Alkyl	
	Ala-204	2.08	Donor-Donor	
L68 vs PDB ID 4EY6	His-447	1.74	Conventional Hydrogen Bond	−5.31
	Trp-86	4.80/5.28	Pi-Alkyl	
	Trp-86	3.42/3.90	Pi-T-Stacked	
	Try-337	2.97	Carbon Hydrogen Bond	
	Glu-202	3.74	Conventional Hydrogen Bond	

**Table 4:** Interactions between the juvenile hormone and the most promising molecules.

Molecular Docking	Residues	Distance (Å)	Type	ΔG (Kcal·mol^−1^)
JHIII vs PDB ID 5V13	Trp-53	4.53/4.95/5.02	Pi-Alkyl	−5.61
	Val-51	4.74	Alkyl	
	Leu-74	4.98	Alkyl	
	Val-68	4.89	Alkyl	
	Tyr-129	5.28/5.80	Pi-Alkyl	
	Tyr-133	5.28/5.29	Pi-Alkyl	
	Tyr-64	5.01	Pi-Alkyl	
	Ile-140	4.98	Alkyl	
	Phe-144	5.00	Pi-Alkyl	
L46 vs PDB ID 5V13	Trp 53	4.12	Pi-Alkyl	−8.46
	Trp 53	2.98	Pi-Lone-Pair	
	Val-51	3.94	Pi-Sigma	
	Val-68	3.93	Alkyl	
	Val-65	3.65	Alkyl	
	Ala-281	4.24	Pi-Alkyl	
	Tyr-64	5.25	Pi-Alkyl	
	Tyr-33	5.07	Pi-Pi-Stacked	
	Tyr-33	4.40	Pi-Alkyl	
L66 vs PDB ID 5V13	Trp-53	4.69/2.40	Pi-T-Stacked	−10.56
	Val-51	4.94	Pi-Alkyl	
	Val-51	3.72	Pi-Sigma	
	Val-68	3.81/5.32	Pi-Alkyl	
	Val-65	3.90	Pi-Alkyl	
	Phe-144	5.24	Pi-T-Stacked	
	Tyr-129	4.95	Pi-T-Stacked	
	Tyr-129	3.23	Pi-donor	
	Tyr-64	2.80/1.90	Conventional Hydrogen Bond	
	Tyr-33	5.69/4.40	Pi-Pi-Stacked	
	Ala-281	3.76	Pi-Sigma	
	Leu-74	5.30	Pi-Alkyl	
L68 vs PDB ID 5V13	Trp-53	3.83	Pi-donor Hydrogen Bond	−8.70
	Pro-55	5.34	Pi-Alkyl	
	Trp-50	2.99	Pi-lone Pair	
	Val-68	3.93	Alkyl	
	Val-65	4.35	Alkyl	
	Tyr-64	4.67	Alkyl	
	Ser-69	2.75	Conventional Hydrogen Bond	

**Table 5:** Data from protocols used in the molecular docking validation.

Receptor	Ligand	Ligand Coordinates of the Grid Center	Grid Size (Points)
AChE (PDB ID 1QON)	9-(3-Iodobenzylamino)-1,2,3,4-tetrahydroacridine	*X* = 33.8687 *Y* = 67.6341 *Z* = 10.1163	74 *x* 33 *y* 18 *z*
AChE (PDB ID 4EY6)	(–)-Galantamine	*X* = 8.2053 *Y* = −60.5991 *Z* = −24.2265	74 *x* 32 *y* 18 *z*
Juvenile hormone (PDB ID 5V13)	Methyl(2*E*,6*E*)-9-[(2*R*)-3,3-dimethyloxiran-2-yl]-3,7-dimethylnona-2,6-dienoate	*X* = -251.7312 *Y* = 9.0631 *Z* = 353.4562	48 *x* 29 *y* 20 *z*
